# Astrocyte-Derived Exosomal miR-148a-3p Suppresses Neuroinflammation and Restores Neurological Function in Traumatic Brain Injury by Regulating the Microglial Phenotype

**DOI:** 10.1523/ENEURO.0336-23.2024

**Published:** 2024-02-08

**Authors:** Yan Qian, Xin Li, Guiliang Li, Huali Liu, Qiaofen Li, Xia Liu, Yang Zhang, Zongying He, Ying Zhao, Hong Fan

**Affiliations:** ^1^Rehabilitation Medicine, Qujing No.1 Hospital, Qujing, Yunnan 655000, China; ^2^Rehabilitation Medicine, The Second Affiliated Hospital of Kunming Medical University, Kunming, Yunnan 650000, China

**Keywords:** exosomes, inflammatory response, microglial phenotype, neurological function, traumatic brain injury

## Abstract

Interactions between astrocytes and microglia play an important role in the regeneration and repair of traumatic brain injury (TBI), and exosomes are involved in cell–cell interactions. A TBI model was constructed in rats. Brain extract (Ext) was isolated 1 d after TBI. Astrocyte-derived exosomes were obtained by coculturing Ext with primary astrocytes, and the morphology of exosomes was observed by electron microscopy. The isolated exosomes were cocultured with microglia to observe phenotypic changes in M1 and M2 markers. Aberrant RNA expression was detected in necrotic brain tissue and edematous brain tissue. The role of miR-148a-3p in regulating microglial phenotype was explored by knocking down or overexpressing miR-148a-3p. Finally, the effect of miR-148a-3p on TBI was studied in a rat TBI model. Astrocyte-derived exosomes stimulated by Ext promoted the transition of microglia from the M1 phenotype to the M2 phenotype. MiR-148a-3p was highly expressed in TBI. Transfecting miR-148a-3p promoted the transition of microglia from the M1 phenotype to the M2 phenotype and inhibited the lipopolysaccharide-induced inflammatory response in pre-microglia. In a rat TBI model, miR-148a-3p significantly improved the modified neurological severity score and attenuated brain injury, which promoted the transition of microglia from the M1 phenotype to the M2 phenotype. MiR-148a-3p alleviated TBI by inhibiting the nuclear factor κB pathway. Astrocyte-derived exosomal miR-148a-3p regulates the microglial phenotype, inhibits neuroinflammation, and restores neurological function in TBI. These results provide new potential targets for the treatment of TBI.

## Significance Statement

This study reported that astrocyte-derived exosomal miR-148a-3p promoted the transition of microglia from the M1 phenotype to the M2 phenotype to suppress neuroinflammation and restore neurological function in traumatic brain injury (TBI). These findings provide a regulatory mechanism based on the interaction of astrocytes and microglia in the development of TBI and new potential targets for the treatment of TBI.

## Introduction

Traumatic brain injury (TBI) is the leading cause of death and disability among adolescents worldwide. It is an externally induced alteration in brain function that is characterized by the loss or impairment of consciousness, loss of memory before and after injury, neurological deficits (e.g., weakness, loss of balance, and altered vision), or mental disturbances such as confusion, disorientation, and slowed thinking (Menon et al., 2010; Peeters et al., 2015; Eme, 2017). Pathologically, brain injury is divided into primary and secondary injury. The inflammatory response, which is a pathological feature of the injured brain, is present in the chronic and acute stages of TBI, and it plays an important role in the transformation from primary to secondary injury (Shojo et al., 2010; Blennow et al., 2012). The inflammatory response is protective and a natural defense, but various stimuli and disordered immune effectors (including proinflammatory cells and chemokines) typically activate neuroinflammation and exacerbate the pathological progression of tissue damage (Piao et al., 2012; Faden and Loane, 2015; Chiu et al., 2016; Simon et al., 2017). Therefore, suppressing excessive inflammation is essential for improving neurological function after TBI.

Macrophages in the central nervous system and microglia are key cells that regulate immune responses to maintain brain homeostasis, normally exhibiting a neuro-specific phenotype (Schmid et al., 2009) and maintaining a relatively quiescent surveillance phenotype to detect brain parenchyma (Davalos et al., 2005; Nimmerjahn et al., 2005). After TBI, microglia in the physiological state are rapidly activated and migrate to the site of injury. Activated microglia produce and release inflammatory mediators and affect peripheral immune cells, including neutrophils, lymphocytes, and macrophages, and the interaction between cells and inflammatory mediators leads to peripheral neuronal overreaction, which greatly exacerbates TBI (Chiu et al., 2016). Recent studies have shown that microglia exert dual effects on TBI. Microglia exhibit an M1 proinflammatory phenotype or an M2 anti-inflammatory phenotype after TBI and release growth factors that repair injured tissue (Harry, 2013; Loane and Kumar, 2016). Notably, modulating the polarization of microglia, especially by promoting M2 phenotypic conversion after TBI, may be a new therapeutic strategy to suppress the excessive inflammatory response after TBI and improve neurological function.

Exosomes are membrane vesicles (40–150 nm in diameter) secreted by various cell types, and there is growing evidence that exosomes act as intercellular messengers to regulate cellular function through an intercellular transfer of biologically active molecules such as proteins, RNA, and miRNA (Colombo et al., 2014; Sluijter et al., 2014; Quek and Hill, 2017). Human bone marrow mesenchymal stem cell–derived exosomes attenuate ischemic reperfusion injury (Liu et al., 2021) and demyelination in the central nervous system (Zhang et al., 2022) during spinal cord injury (Fan et al., 2021).^.^ The induction of neuroinflammation and apoptosis is achieved by modulating the M1/M2 phenotype of microglia, which can also suppress neuroinflammation. Astrocytes and microglia actively participate in various pathological conditions, including TBI. Activated astrocytes produce many regulatory factors that affect CNS immunity and provide negative feedback to activated microglia (Min et al., 2006; Farina et al., 2007). Astrocyte recruitment was impaired in a TBI mouse model when the Cdc42 gene was deleted, and an increase in the number of microglia was observed in regions with a reduced astrocyte population (Robel et al., 2011). Furthermore, astrocytes may act as controllers to rapidly inhibit microglial activation in the injured brain (Kim et al., 2010). Astrocyte-derived exosomes are powerful mediators of neuronal plasticity, immune responses, and neuronal survival in a variety of pathological conditions (Lafourcade et al., 2016). Astroglial-derived exosomes play an important role in protecting neurons from oxidative stress, including the regeneration and repair of TBI or related diseases, but little is known about the mechanisms by which they activate microglia and their role in mediating neuroinflammation after TBI.

The present study investigated the effect of astrocyte-derived exosomes on microglial phenotypes in an in vitro TBI model and examined exosome-encapsulated functional miRNAs in TBI rats in vivo. The results provide new support for astrocyte-mediated regulation of microglia and a potential therapeutic target for neuroinflammation in brain injury via this regulatory mechanism.

## Materials and Methods

### TBI animal model and experimental groupings

Male Sprague Dawley (SD) rats (age, 8–10 weeks; body weight, 220–250 g) were purchased from Hunan SJA Laboratory Animal. The animals were housed in a controlled environment (temperature, 20–25°C, 12 h light/dark cycle), and the TBI model was induced according to a previous report (Jassam et al., 2017). Briefly, deformational brain injury was induced via craniotomy in previously anesthetized animals using pneumatic or electromechanically controlled cortical impact (CCI) devices. All rats in the TBI group showed loss of motor ability in the injured hindlimb, unilateral hemiplegia, and decreased muscle tone below the injury plane. The injured hind limbs did not respond to acupuncture, and the animals could not walk in a straight line, which indicated that TBI modeling was successful. The rats were randomly divided into four groups: the sham group (*n* = 20), TBI group (*n* = 20), TBI + miR-148a-5p agomir group (*n* = 20), and TBI + miR-148a-5p antagomir group (*n* = 20). On Days 1, 3, and 7, the rats were killed (*n* = 6), tissue around the traumatic area of the injured brain was collected, and similar areas were collected from the sham rats for subsequent experiments. The cortex was collected for immunofluorescence analysis, qRT-PCR, and Western blotting. All animal experimental protocols were approved by the Animal Experiment Ethics Committee of Kunming Medical University, and the animal procedures adhered to the ARRIVE guidelines 2.0.

### Primary microglia and astrocyte culture

Primary microglia and astrocytes were obtained and purified from postnatal SD rats (Long et al., 2020). Briefly, newborn SD rats were anesthetized via passive inhalation of 2% isoflurane, and the cerebral cortex was collected and sectioned after the rats were decapitated. The tissue was dissociated in trypsin-EDTA (0.25%; Beyotime), filtered, and centrifuged. The supernatant was discarded, and the cells were added to culture flasks supplemented with a medium containing 10% fetal bovine serum. After 8–10 d of incubation, microglia were removed from the astrocyte monolayer by shaking at 220 rpm for 40 min. The medium was added to 24-well plates and shaken continuously at 220 rpm for 18 h to remove oligodendroglia, and the remaining cells in the flask were astrocytes with >90% purity. Astrocytes and microglia were collected for a subsequent experiment.

### Cell transfection and intracerebroventricular infusion

The miR-148a-3p mimic, negative control mimic (NC mimic), and agomir were constructed by Shanghai Genetic Chemistry. Microglia were transfected with 100 nM miR-148a-3p mimics or NC mimics according to the manufacturer’s protocol (Thermo Fisher Scientific). The miR-148a-3p agomir was mixed and incubated at room temperature for 15 min for lateral ventricular injection. A single dose of the miR-148a-3p agomir (5 nmol, 20 µl) or miR-148a-3p antagomir (5 nmol, 20 µl) was injected into the lateral ventricle 20 min after CCI (Long et al., 2020).

### Brain extract preparation

Brain extract (Ext) was prepared as previously described (Chen et al., 2002). Briefly, TBI was induced in the rats, and the cortex was collected 1 d later. The collected cortical tissues were placed in glass tubes containing a complete culture medium. Glass grinding rods were used, and the ground tissue was centrifuged (1,000 rpm for 10 min). The supernatant was collected and stored in 1.5 ml electropolished tubes at −80°C.

### Isolation of astrocyte-derived exosomes and transmission electron microscopy

Primary astrocytes were incubated with TBI brain extract (10 µg/ml) for 48 h. As described in the literature (Xin et al., 2012), supernatants collected from cultured astrocytes were centrifuged at 4°C (3,000 rpm for 10 min) to remove suspended cells or debris, and the supernatant was stored at 4°C for use as a blank exosome control. Samples were homogenized on a vortex shaker and centrifuged at 100,000 × *g* for 60 min at 4°C to obtain exosome pellets. The pellets were resuspended in 50 µl of PBS and stored at −80°C for use. Exosome morphology was observed by transmission electron microscopy (JEM-1200EX, JEOL).

### Exosome labeling and uptake

Exosomes secreted from astrocytes were fluorescently labeled using the lipophilic dye PKH26 (Sigma-Aldrich). Exosomes were resuspended in diluent solution and PKH26 and incubated at room temperature for 5 min. PKH26-labeled exosomes were diluted with PBS and ultracentrifuged at 150,000 × *g* at 4°C for 60 min to remove the unbound dye. PKH26-labeled exosomes were incubated with primary microglia for 24 h. Then, the cells were fixed and stained with DAPI. The cells were visualized using laser scanning confocal microscopy (DMi8, Leica), and photographic images were obtained.

### Western blot analysis

Protein concentrations of medium supernatants, cells, and animal tissues were determined using a BCA kit (Beyotime), and the proteins were subjected to 10% SDS-PAGE. The separated proteins were transferred to nitrocellulose membranes. The membranes were incubated with primary antibodies against Arg1 (1:1,000, ab315110), iNOS (1:1,000, ab178945), TNF-α (1:1,000, ab307164), Iba-1 (1:1,000, ab178846), CD9 (1:1,000, ab307085), CD63 (1:1,000, ab108950), CD206 (1:1,000, ab300621), IL-4 (1:500, ab9811), IL-10 (1:1,000, ab9969), ERK (1:10,000, ab184699), phospho-ERK (1:10,000, ab201015), nuclear factor (NF)-kB p65 (1:1,000, ab16502), and phospho-NF-kB p65 (1:1,000, ab76302) overnight at 4°C. The membranes were incubated with a blocking solution and labeled with the corresponding secondary antibodies. The membranes were washed three times, and images were scanned using a Tanon 5200 imaging system after ECL chemiluminescence treatment.

### Neurological function score

TBI rat neurological severity score [modified neurological severity score (mNSS)] assessments were performed before CCI and on Days 1, 3, and 7 after CCI. The severity scores were graded on a scale from 0 to 18, where 0 represented the normal score and 18 represented the maximum deficit score. mNSS assessments included muscle status; abnormal movements; visual, tactile, and proprioceptive sensations; reflex responses; and balance tests. Higher scores indicated more severe injury.

### Determination of brain water content

According to the reference (Wei et al., 2015), the animals were killed 7 d after TBI, and brain tissue was collected and weighed (wet weight). Brain tissues were dried at 80°C for 72 h and weighed again (dry weight). Brain water content (%) was calculated using the following formula: (wet weight − dry weight)/wet weight × 100%.

### qRT-PCR

Total RNA was extracted from microglia or tissues using TRIzol reagent (Invitrogen), and the concentration was determined. Real-time PCR was performed to analyze miRNA using kits and instruments according to the manufacturer’s protocols. mRNA and miRNA expression were measured using SYBR Premix Ex Taq TM II (Takara) on an ABI-Prism 7500 real-time PCR system (Applied Biosystems). Gene expression was analyzed using the 2^−ΔΔCt^ method. All primer sequences are shown in Table 1.

**Table 1.** Primer sequences

Gene sequence	Primer name	Sequence (5′ → 3′)
iNOS	Forward	CAGGGTGTTGCCCAAACTG
	Reverse	GGCTGCGTTCTTCTTTGCT
TNF-α	Forward	TCTTCTCATTCCTGCTTGTGG
	Reverse	GGTCTGGGCCATAGAACTGA
Arg-1	Forward	AGACAGCAGAGGAGGTGAAGTAC
	Reverse	GGTAGTCAGTCCCTGGCTTATGGT
IL-10	Forward	ACCTGGTAGAAGTGATGCCC
	Reverse	ACACCTTGGTCTTGGAGCTT
IL-4	Forward	GTAGGGCTTCCAAGGTGCTTC
	Reverse	CATGATGCTCTTTAGGCTTTCCAG
RTN4	Forward	AGTACTTACGAAAGAAGCAGG
	Reverse	GTATCACAGGCTCAGATGCAG
GAPDH	Forward	ACAACTTTGGTATCGTGGAAGG
	Reverse	GCCATCACGCCACAGTTTC
IL-1β	Forward	TGCAGAGTTCCCCAACTGGTACA
	Reverse	GTGCTGCCTAATGTCCCCTT-G
miR-148a-3p	Forward	GAGACACTCCGACTCTGAGT
	Reverse	GTTCTGTAGTGCACTGAC
U6	Forward	CTCGCTTCGGCAGCACA
	Reverse	AACGCTTCACGAATTTGCGT
IL-6	Forward	CCGGAGAGAGACTTCACAG
	Reverse	CATTTCCACGATTTCCCAGA
β-actin	Forward	TGGAATCCTGTGGCATCCATGAAAC
	Reverse	TAAAACGCAGCTCAGTAACAGTCCG
CD206	Forward	TCTTTGCCTTTCCCAGTCTCC
	Reverse	TGACACCCAGCGGAATTTC
CD32	Forward	CCAGAAAGGCCAGGATCTAGTG
	Reverse	GGGAACCAATCTCGTAGTGTCTGT

### Immunofluorescence staining

Cells and tissues were incubated with primary antibodies, including Arg1 (1:5,000, ab315110), Iba1 (1:1,000, ab178846), GFAP (1:5,000, ab7260), iNOS (1:100, ab178945), and CD9 (1:250, ab307736), overnight at 4°C, washed with PBS, and incubated with the conjugated secondary antibodies for 1 h at room temperature in the dark. Cell nuclei were stained with DAPI. Immunofluorescence signals were observed using an Olympus FluoView laser scanning confocal microscope.

### Statistical analysis

All experiments were performed in triplicate. Statistical analysis was performed using GraphPad Prism 8 (GraphPad Software). The results are expressed as the mean ± standard deviation (SD). Two groups were compared with Student’s *t* test, and three or more treatments or groups were compared with one-way ANOVA followed by Tukey–Kramer *post hoc* analysis. A *p* value of <0.05 was defined as statistically significant.

## Results

### Astrocytes release exosomes after stimulation with brain extracts

**Figure 1.**
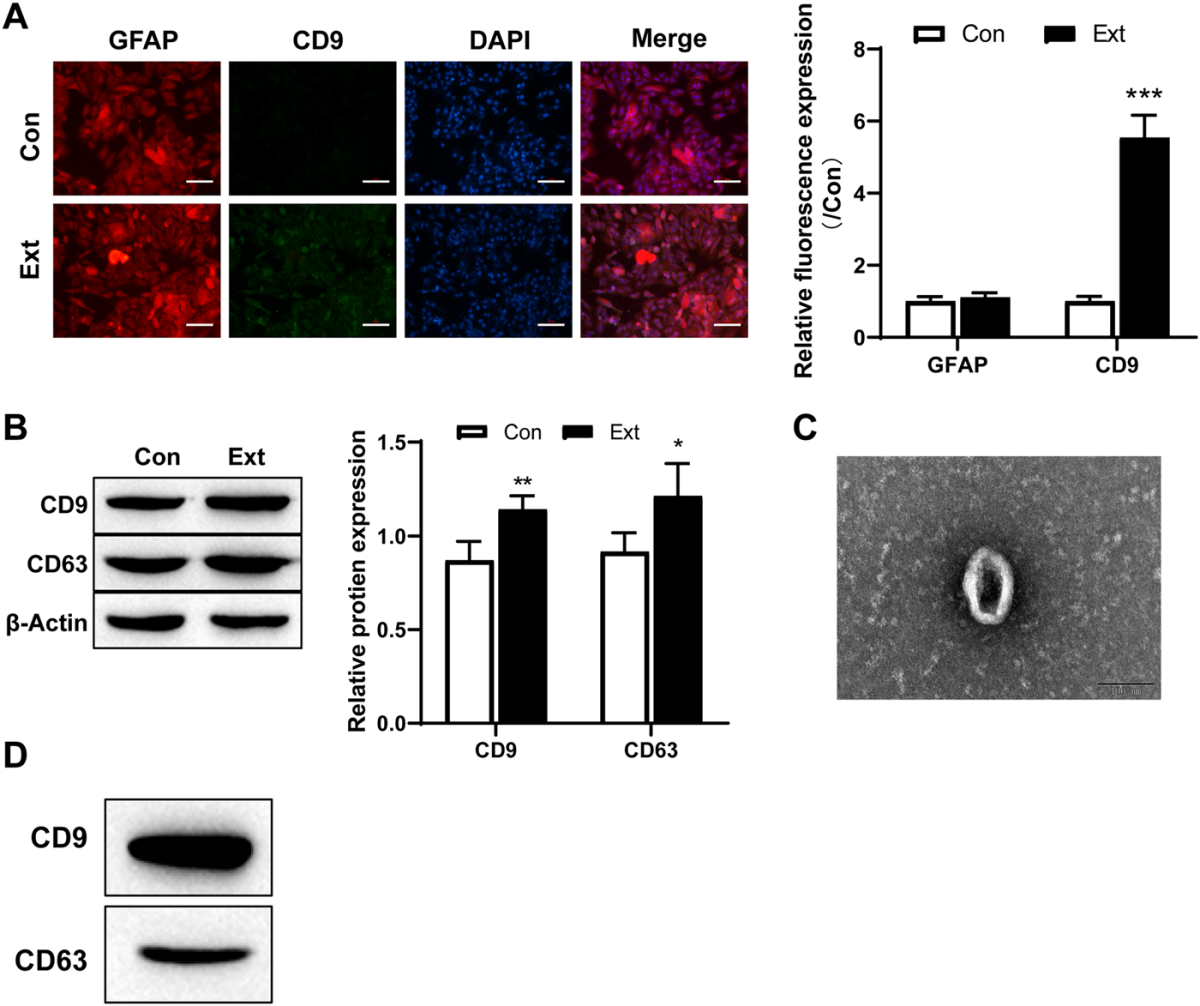
Astrocytes release exosomes after stimulation with brain extracts. ***A***, Exosomes derived from astrocytes were increased after brain extract stimulation (scale bar = 100 µm). ***B***, The expression of the exosomal markers CD9 and CD63 in the medium of primary cultured astrocytes was detected by Western blotting after 24 h of brain extract treatment (*n* = 3, *t* test). ***C***, A representative TEM image of exosomes in the culture medium of stimulated astrocytes after 24 h of brain extract treatment (scale bar = 100 nm). ***D***, The expression of the exosomal markers CD9 and CD63 in astrocyte-derived exosomes was detected by Western blotting. Con, control; Ext, brain extracts. Compared with the Con group, *p < 0.05 and **p < 0.01.

Astrocyte-derived exosomes can mediate TBI. We examined the expression of exosomes using immunofluorescence staining and Western blotting, and the results showed a significant increase in the expression of CD9 and CD63 after stimulation with TBI brain extract (Fig. 1*A,B*). Exosomes were isolated from astroglia and observed by using electron microscopy. TEM imaging showed that exosomes derived from astrocytes had a typical cup-shaped morphology and were within the range of 100 nm (Fig. 1*C*). The exosome markers CD9 and CD63 were also detected (Fig. 1*D*).

### TBI-injured astrocyte-derived exosomes are taken up by primary microglia and promote M2 microglial polarization

**Figure 2.**
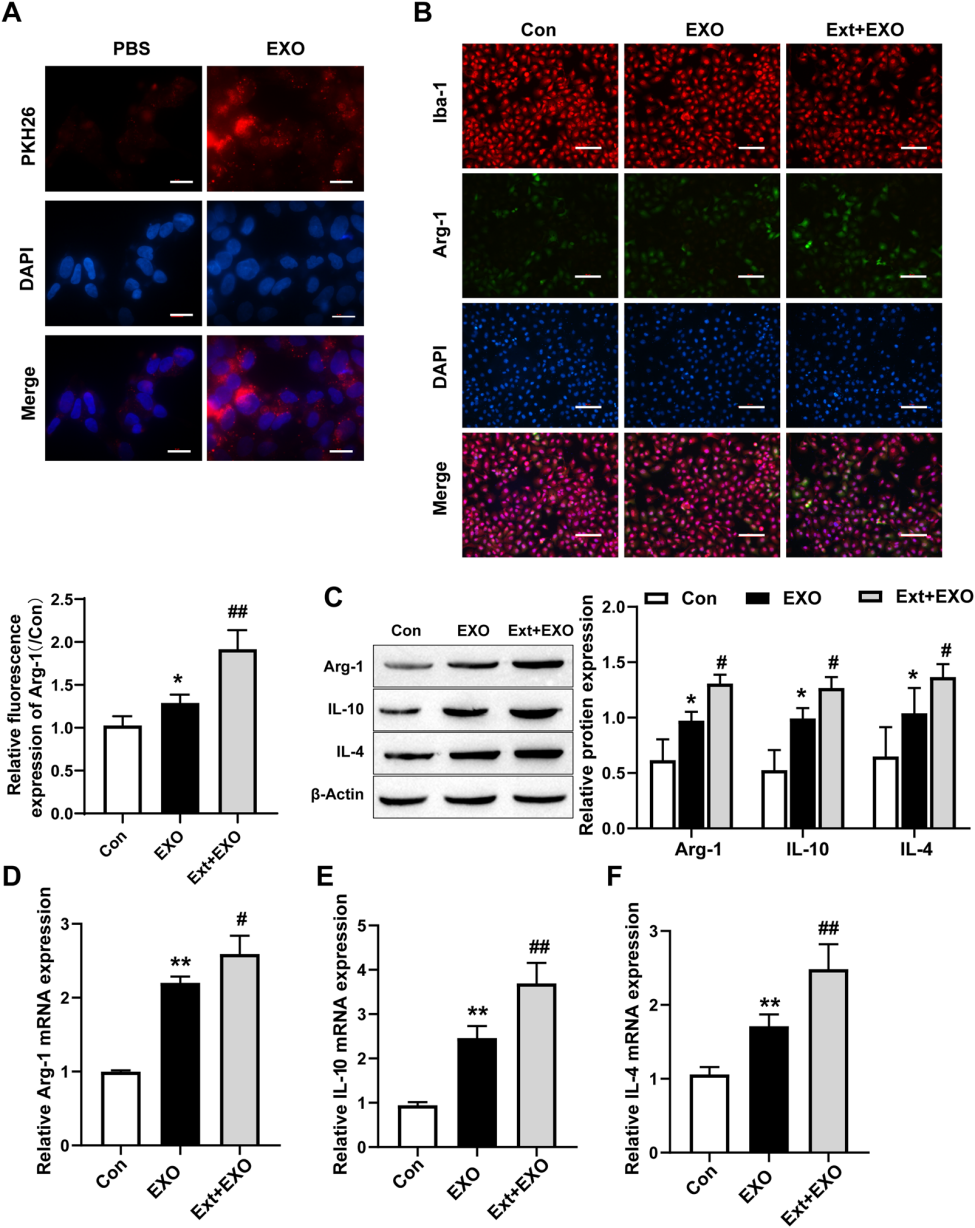
TBI-injured astrocyte-derived exosomes are taken up by primary microglia and promote M2 microglial polarization. ***A***, PKH26 dye was used to assess whether exosomes were taken up by microglia (scale bar = 20 µm). ***B***, Fluorescence confocal microscopy images showed increased expression of Iba-1 (microglia markers) and Arg-1 (M2 marker) after treatment with astrocyte-derived exosomes (scale bar = 100 µm). ***C***, Arg-1, IL-4, and IL-10 (M2 markers) were detected by Western blot analysis (*n* = 3, one-way ANOVA). ***D–F***, The mRNA expression of Arg-1, IL-4, and IL-10 (M2 markers) was detected by qRT-PCR (*n* = 3, one-way ANOVA). Exo, exosomes incubated with microglia for 4 h. Ext + Exo, primary microglia stimulated with brain extracts for 24 h and incubated with exosomes for 4 h. Compared with the Con group, *p < 0.05 and **p < 0.01. Compared with Con + Exo, ^#^*p* < 0.05 and ^##^*p* < 0.01.

To investigate the effect of astrocyte-derived exosomes on microglial phenotype, microglia were treated with TBI-injured astrocyte exosomes. PKH26-labeled exosomes were successfully taken up by microglia (Fig. 2*A*). We detected Iba-1 (microglia marker) and Arg-1 (M2 marker) by using immunofluorescence; the results showed that exosomes from astrocytes could significantly promote the conversion of microglia to the M2 phenotype, and exosomes from astrocytes could further promote this transformation compared with astrocyte-derived exosomes from normal sources of brain tissue extract (Fig. 2*B–F*). These data suggested that astrocyte-derived exosomes promoted M2 microglial polarization.

### MiR-148a-3p is highly expressed in astrocytes after TBI

**Figure 3.**
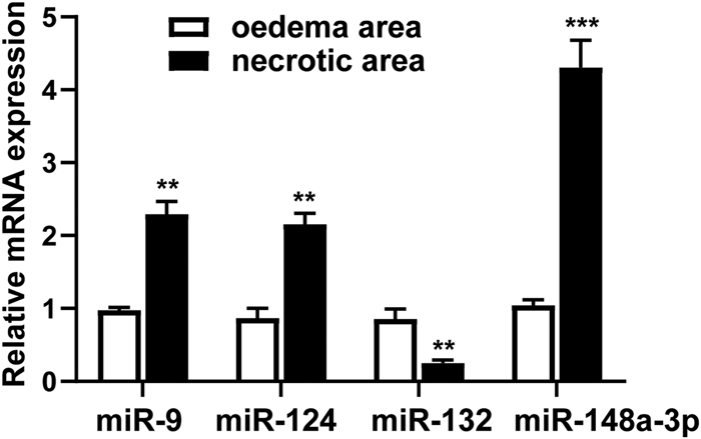
The expression of miR-873a-5p in necrotic brain tissue and edematous brain tissue in TBI was measured by qRT-PCR. Brain tissue samples from necrotic and edematous areas were collected 3 d after TBI. Compared with the edema group, **p < 0.01 and ***p < 0.001, *n* = 3, *t* test.

Exosomes are rich in miRNAs and can deliver miRNAs from host cells to target cells to further regulate the function of recipient cells. To examine the neuroprotective mechanisms of exosome-derived from astrocytes, we examined several miRNAs associated with neuroprotection, including miR-9, miR-124, miR-132, and miR-148a-3p. The qRT-PCR results showed that miR-9 (compared with the edema group, 2.4-fold), miR-124 (2.3-fold), and miR-148a-3p (4.1-fold) expression was upregulated in the necrotic area, and miR-132 expression was downregulated. Among the upregulated miRNAs, abnormal expression of miR-148a-3p (4.1-fold) was more obvious (Fig. 3). Therefore, we chose miR-148a-3p as the research focus.

### Effect of miR-148a-3p on LPS-induced primary microglia

**Figure 4.**
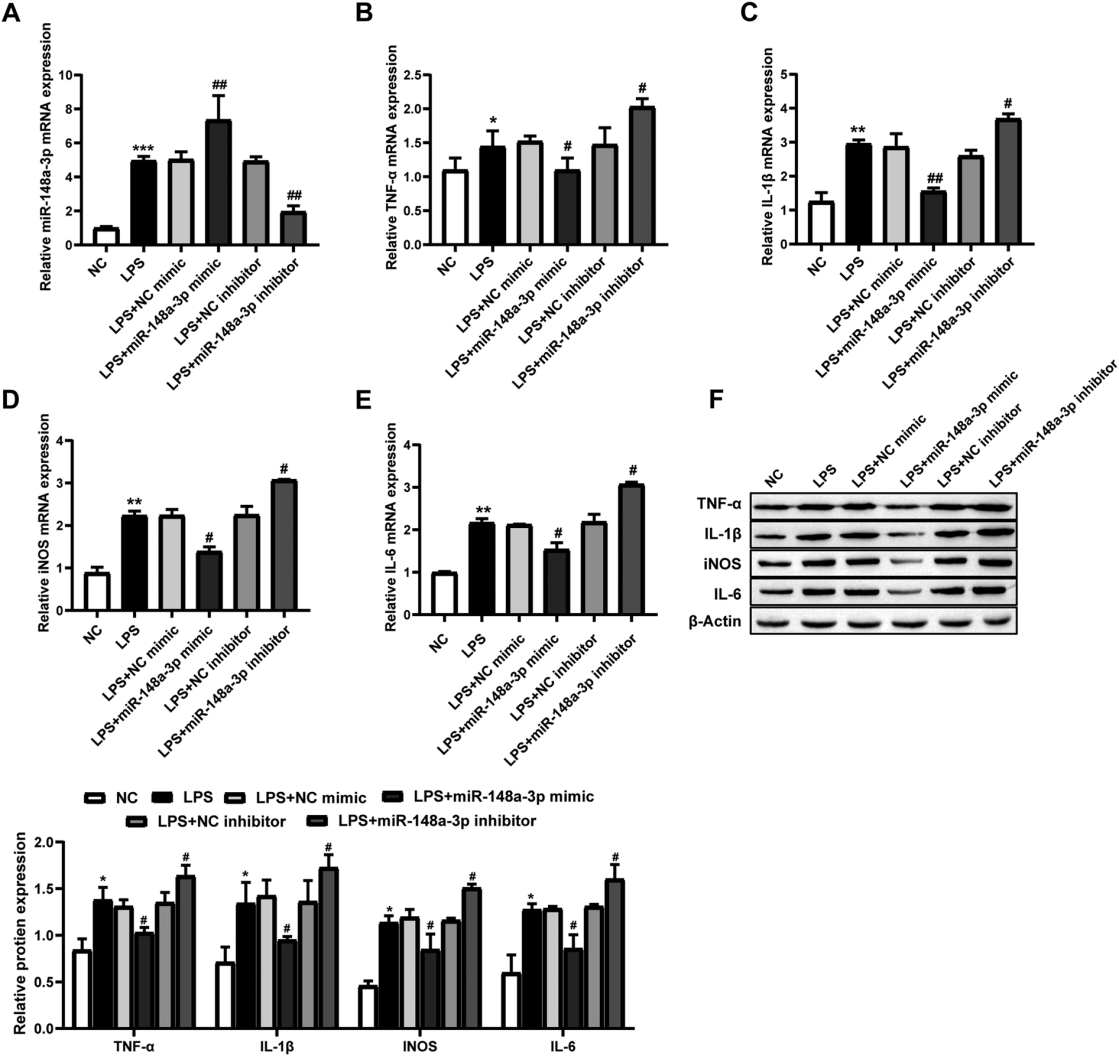
Effect of miR-148a-3p on LPS-induced primary microglia. ***A***, The transfection efficiency of the miR-148a-3p mimic or miR-148a-3p inhibitor was measured by qRT-PCR (*n* = 3, one-way ANOVA). ***B–E***, Western blot analysis of the protein expression of the proinflammatory factors TNF-α, IL-1β, iNOS, and IL-6 (*n* = 3, one-way ANOVA). ***F***, qRT-PCR analysis of the mRNA expression of the proinflammatory factors TNF-α, IL-1β, iNOS, and IL-6 (*n* = 3, one-way ANOVA). LPS (1 µg/ml, 24 h) was used to induce microglial injury. Compared with the NC group, *p < 0.05, **p < 0.01, and ***p < 0.001. Compared with LPS, ^#^*p* < 0.05 and ^##^*p* < 0.01.

To further examine the function of miR-148a-3p, a miR-148a-3p mimic or miR-148a-3p inhibitor was transfected into primary microglia, which were then stimulated with LPS (1 µg/ml, 24 h). MiR-148a-3p expression was measured using qRT-PCR to show successful transfection of the miR-148a-3p mimic or miR-148a-3p inhibitor (Fig. 4*A*). Western blot and qRT-PCR were used to detect the expression of IL-1β, IL-6, TNF-α, and iNOS. The results showed that compared with the LPS group, the LPS + miR-873a-5p mimic group had significantly reduced expression of the proinflammatory factors IL-1β, iNOS, TNF-α, and IL-6, while the LPS + miR-873a-5p inhibitor group had significantly increased expression of these factors (Fig. 4*B–F*). Taken together, these results suggest that miR-148a-3p inhibits the expression of proinflammatory factors and promotes M2 phenotypic transformation in microglia.

### MiR-148a-3p attenuates brain injury in rats after TBI

**Figure 5.**
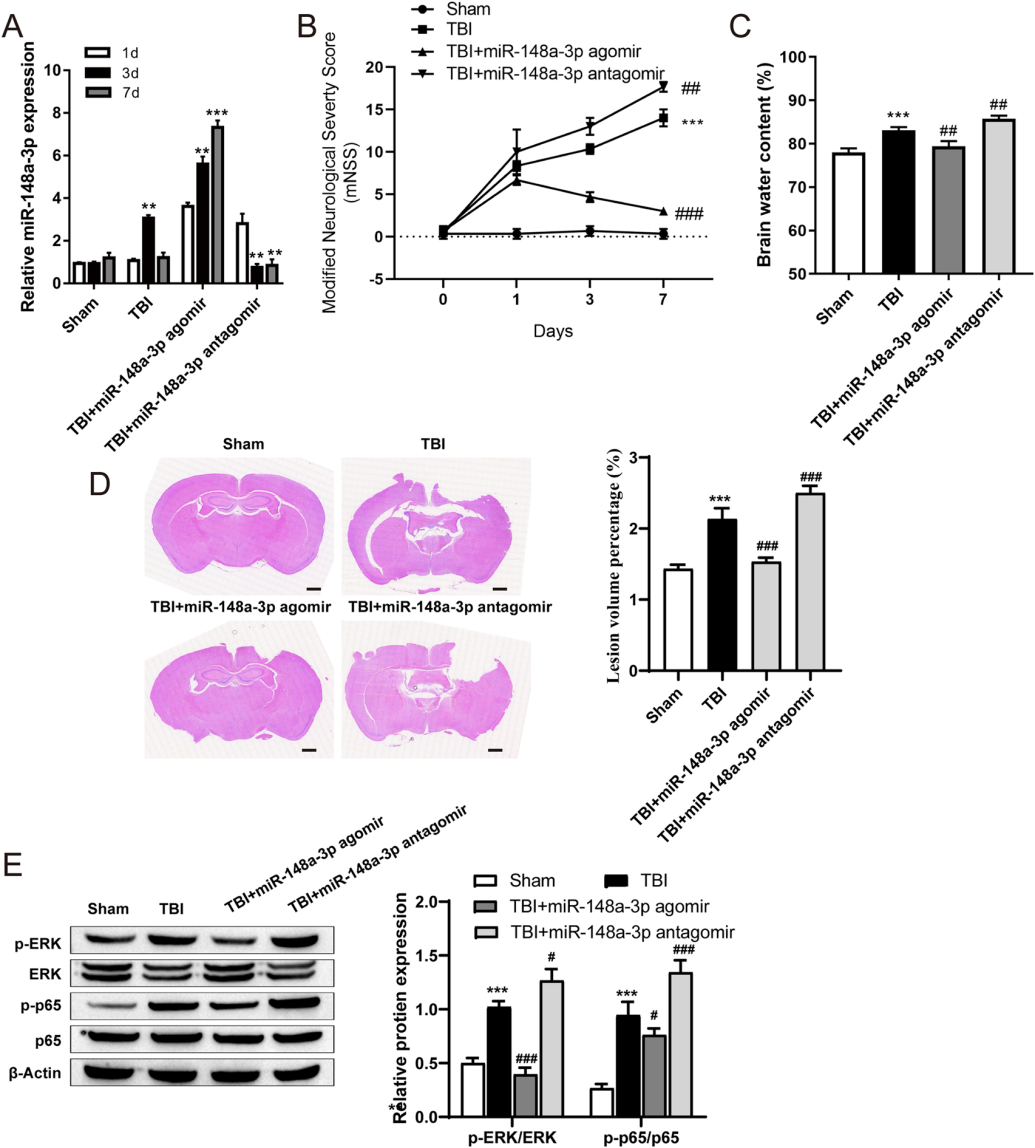
Brain defect area, brain edema, and neurological deficits in rats after miR-148a-3p–mediated attenuation of TBI. ***A***, MiR-148a-3p expression in the cortex was detected by qRT-PCR 1, 3, and 7 d after TBI (*n* = 6, one-way ANOVA). ***B***, Neurological function of rats 1, 3, and 7 d after TBI was assessed by the mNSS (*n* = 6, one-way ANOVA). ***C***, The brain water content was measured by the wet and dry method (*n* = 6, one-way ANOVA). ***D***, The area of the brain defect was observed by HE staining (scale bar = 500 µm). E: Levels of phosphorylated ERK and NF-ΚB P65 were measured by Western blotting 7 d after TBI (*n* = 6, one-way ANOVA). Compared with the sham group, **p < 0.01 and ***p < 0.001. Compared with TBI, ^#^*p* < 0.05, ^##^*p* < 0.01, and ^###^*p* < 0.001.

To identify the effects of miR-873a-5p on TBI, TBI was induced in rats by CCI, after which the miR-873a-5p agomir (mimic) or antagomir (inhibitor) was injected into the lateral ventricle. The expression of miR-148a-3p was detected using qRT-PCR 1, 3, and 7 d after TBI. The expression of miR-148a-3p was increased after TBI compared with that in the sham group and was gradually decreased 7 d after TBI. TBI rats treated with the miR-148a-3p agomir had sustained high miR-148a-3p expression. However, the miR-148a-3p antagomir significantly reduced the expression of miR-148a-3p (Fig. 5*A*). To investigate the neurorestorative effects of miR-148a-3p, we measured the extent of brain edema on Day 7 after TBI and determined the mNSS. The results showed that the area of brain injury, the degree of brain edema, and the mNSS score increased after TBI. However, on the 7th day after treatment with miR-148a-3p, the area of brain injury, the degree of brain edema, and the mNSS score were significantly decreased after TBI. The miR-148a-3p antagomir significantly aggravated brain injury (Fig. 5*B–D*). In addition, we evaluated ERK and NF-κB p65 expression to examine the effect of miR-148a-3p on the NF-κB signaling pathway after TBI. Brain tissue was collected 7 d after TBI. Compared with those in the sham group, the levels of phosphorylated ERK and NF-ΚB P65 were substantially increased in brain tissues collected from TBI rats. MiR-148a-3p inhibited the levels of phosphorylated ERK and NF-ΚB P65, and the miR-148a-3p antagomir exerted the opposite effects (Fig. 5*E*).

### MiR-148a-3p suppresses the inflammatory response by promoting M2 polarization in microglia after TBI in rats

**Figure 6.**
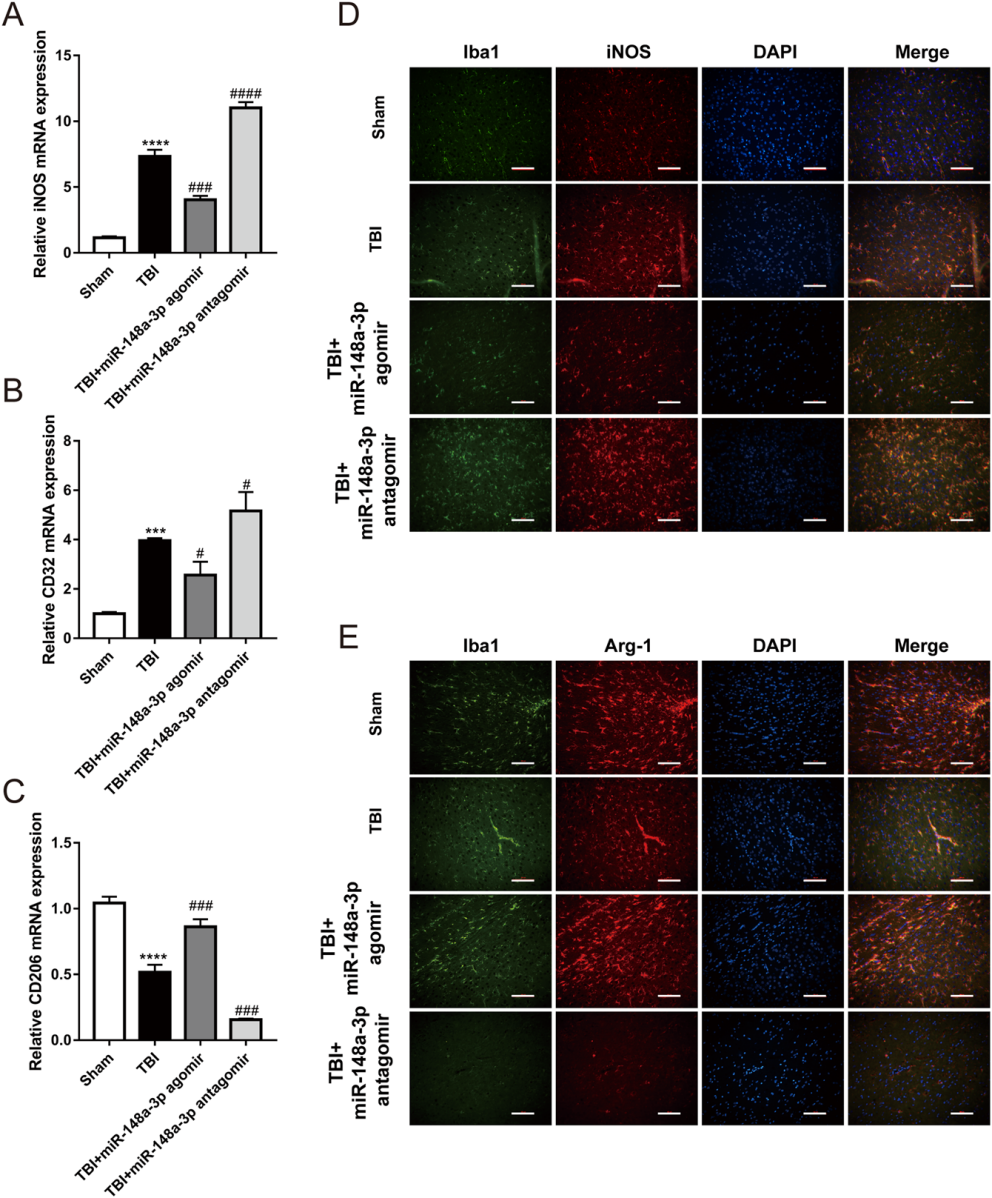
MiR-148a-3p suppresses the inflammatory response by promoting M2 polarization in microglia after TBI. ***A–C***, The expression of the M1 signature genes iNOS and CD32 and the M2 signature gene CD206 was detected by qRT-PCR in the rat cortex (*n* = 6, one-way ANOVA). ***D***, Immunofluorescence analysis of the expression of the M1 microglial marker iNOS in the rat cortex (scale bar = 100 µm). ***E***, Immunofluorescence analysis of the expression of the M2 microglial marker Arg1 in the rat cortex (scale bar = 100 µm). Compared with the sham group, ***p < 0.001. Compared with TBI, ^#^*p* < 0.05 and ^###^*p* < 0.001.

To assess the effect on microglial polarization after TBI, the expression of M1 phenotypic marker genes (iNOS and CD32) and an M2 phenotypic marker gene (CD206) were examined. The qRT-PCR results showed that the expression of the M1 signature genes CD32 and iNOS and the expression of the M2 signature gene CD206 were significantly increased in the cortex after TBI. MiR-148a-3p agomir treatment significantly decreased CD32 and iNOS expression and increased CD206 expression, and miR-148a-3p antagomir treatment had the opposite effects (Fig. 6*A–C*). We also detected the expression of a microglial marker (Iba1), an M1 phenotypic marker (iNOS), and an M2 phenotypic marker (Arg1) by immunofluorescence analysis. Compared with that in the sham group, the expression of iNOS was increased in the TBI group. MiR-148a-3p agomir treatment inhibited the expression of iNOS, and miR-148a-3p antagomir treatment further increased the expression of iNOS (Fig. 6*D*). Compared with that in the sham group, the expression of Arg1 was downregulated in the TBI group. MiR-148a-3p agomir treatment increased the expression of Arg1, and miR-148a-3p antagomir treatment further inhibited the expression of Arg1 (Fig. 6*E*). These results indicate that treatment with miR-148a-3p promotes the conversion of microglia to the M2 phenotype.

## Discussion

TBI is one of the most common neurological disorders with high mortality and morbidity (Dabin et al., 2022). However, there is still no clinically effective treatment to improve cognition or promote recovery, and it is not clear which factors contribute to cognitive recovery after TBI. This study explored the role of miR-148a-3p in TBI and the mechanism by constructing a rat model of TBI and isolating primary astrocytes and microglia in vitro.

Exosomes are novel diagnostic markers of human diseases and ideal drug carriers. A variety of cell-derived exosomes are involved in brain injury-related diseases (Jiang et al., 2019). Astrocytes and microglia play important roles in TBI. Astrocytes can robustly regulate microglial activation. Astrocyte-derived exosomal miR-92b-3p ameliorates oxygen glucose deprivation–induced apoptosis in neurons (Xu et al., 2019). Astrocyte-derived exosomal miR-7 leads to the downregulation of neuronal/glial 2 and ultimately to synaptic changes after HIV-1 infection (Hu et al., 2020). The present study isolated exosomes from brain extract-stimulated astrocytes in the context of TBI. TEM imaging showed that exosomes derived from astrocytes were mostly within the range of 100 nm. Microglia were converted to the M2 phenotype after treatment with exosomes.

MicroRNAs (miRNAs, approximately 20 nt) are small noncoding single-stranded RNA molecules that regulate target genes by binding to the 3′ untranslated region (3′ UTR). There is increasing evidence that microRNAs play an important role in TBI. For example, Huang et al. (2018) found that an increase in miR-124-3p in microglial exosomes inhibits neuronal inflammation and contributes to neurite outgrowth. MiR-124-enriched exosomes promoted M2 polarization in microglia and enhanced hippocampal neurogenesis after TBI by inhibiting the TLR4 pathway (Yang et al., 2019). In this study, we found that miR-148a-3p expression was upregulated in the necrotic area in comparison with the edematous brain. MiR-148a-3p was enriched in astrocyte-derived exosomes. Overexpression of miR-148a-3p inhibited the expression of proinflammatory factors and promoted M2 polarization in LPS-induced microglia. In vivo experiments exhibited similar results.

Microglia are resident immune cells in the central nervous system and the first responders to neurological changes (Kwon and Koh, 2020). The activation of microglia in transient focal cerebral ischemia may reflect the severity of injury (Emmrich et al., 2015) and is accompanied by morphological and phenotypic changes (Guruswamy and Elali, 2017). The M1 phenotype exerts proinflammatory effects, and the M1 phenotype-specific marker is ion calcium-binding bridging molecule 1 (Iba1) (Patel et al., 2013). The M2 phenotype exerts anti-inflammatory effects and has the specific markers arginase 1 (Arg-1), IL-10, and IL-4 (Estévez et al., 2006). The present study showed that astrocyte-derived exosomes inhibited microglial M2 phenotypic conversion and suppressed the inflammatory response. In TBI rats, the area of brain injury, the degree of brain edema, and the mNSS score were increased. However, on the 7th day after treatment with miR-148a-3p, the area of brain injury, the degree of brain edema, and the mNSS score were significantly decreased after TBI. In addition, the Western blot results showed that miR-148a-3p attenuated TBI by inhibiting the NF-κB pathway.

In conclusion, our study showed that astrocyte-derived exosomal miR-148a-3p promoted neurological recovery from TBI by suppressing the cellular inflammatory response and promoting M2 polarization in microglia. These findings provide a regulatory mechanism based on the interaction of astrocytes and microglia in the development of TBI, which may be a new target for the treatment of TBI.

## Availability of data and materials

The datasets used and/or analyzed in the current study are available from the corresponding author upon reasonable request.

## Synthesis

Reviewing Editor: Viji Santhakumar, University of California Riverside Decisions are customarily a result of the Reviewing Editor and the peer reviewers coming together and discussing their recommendations until a consensus is reached. When revisions are invited, a fact-based synthesis statement explaining their decision and outlining what is needed to prepare a revision will be listed below. The following reviewer(s) agreed to reveal their identity: Nicole Schartz.

The manuscript examines the influence of astrocytes on microglia activation by utilizing exosomes derived from astrocytes that are injured by TBI. These exosomes express high levels of miRNA-148a-3p, and when co-cultured with microglia, exosomes from TBI astrocytes promote microglial activity. miRNA-148a-3p treatment in microglia that undergo LPS stimulation inhibits expression of inflammatory genes/proteins. This study provides insightful information on the mechanisms by which astrocytic activation attenuates disease-enhancing or pro-inflammatory microglial responses after TBI and on how astrocytes and microglia communicate. In this manuscript, authors show that astrocyte exosome contains miR-148a-3p. Next, they show that miR-148a-3p can inhibit LPS induced microglia activation and M2 microglia activation after TBI. Further, they found that miR-148a-3p targets RTN4 and regulates RTN4 expression. miR-148a-3p can also reduce brain damage and edema after TBI.

While the work is novel and interesting to TBI field lack of detailed methods, appropriate discussion of statistics and low image quality make it difficult to adequately assess the validity of the data presented.

Major Concerns

1. The methods are not adequately described. The organization of the methods and the level of description should be modified to make the experiments more understandable. Perhaps it would be beneficial to describe all the animal manipulations first and then describe the isolation of exosomes from these animals, then the culture of primary microglia and astrocytes. A very thorough re-write of the methods is recommended.

a. Essential methodological details such as how traumatic brain injury (TBI) was conducted and validated by histology need to be provided. When was the brain tissue extracted after TBI in Fig.3 ?

b. The LPS experiments are completely absent from the methods section. What concentration of LPS used and how long was the treatment with LPS in fig 4?

c. The methods section of the abstract could use more detail so the reader can understand the order of experiments and which samples (tissue or cultured cells) were manipulated in the different ways, instead of only listing the techniques used.

d. Clarify whether rat or mouse was used- line 12 and 86, authors say they used a mouse model, but all methods only mention rat

e. Line 185-186: Dilution of antibodies for IHC is not shown. Figure 2 also shows staining for phalloidin but that antibody is not listed.

f. Section 3.6 (starting at line 297): it was not made clear in the methods that rats were sacrificed at 1, 3, and 7 days. It is implied in the methods (line 162) that neurological scores were taken over time, but this results section implies that rats were sacrificed at each neurological score day. This needs to be clarified in the methods and results. How many rats were sacrificed at each day (total N per group is 20, but it is not specified how many for each day)? What tissue was assessed 1-7 days after TBI with PCR? The methods only say microglia. Were microglia purified from these samples? All of this should be clarified in the methods and results.

g. Line 218: The authors state that they investigated the effects of astrocyte-derived exosomes on the activation of TBI-injured microglia in the results, but this is not clearly explained in the methods. As the methods state, primary astrocyte and microglia cultures were purified from postnatal rats, and astrocyte exosomes were purified from TBI-injured rats. Would a better description be that "microglia were treated with TBI-injured astrocyte exosomes"? If microglia were derived from TBI-rats or if microglia in culture were subjected to a "TBI"-like inflammatory condition, it should be stated in the methods.

2. Statistical methods are not adequately detailed and not included in figure legends. This should be addressed.

3. Figure quality is poor and needs to be addressed. Scale bars in figures and statistical test details in legends need to be included.

a. In Fig.1A, scale bar is missing or hard to identify. Scale bar is not listed in figure legend.

b. Fig1B, what is the sample size? What was the statistical test used?

c. Fig2 A-B, the image quality is too low to identify microglia.

d. Fig5. Unclear what tissue or cells were used for the experiments: microglia or astrocyte or brain tissue?

e. Fig.6 Authors need to include representative images of how damaged brain tissues look like in experimental groups. IBA1 staining needs to be included to show microglia morphology under different condition.

f. Fig7. Image quality needs to be improved. Unclear if this data is from cell culture or brain tissue?

4. Fig. 1: Are astrocyte exosomes derived from brain extracts or control culture. Is a difference expected between con and ext preparations? Authors should consider quantifying percent field area.

5. Fig. 2: the authors state that the number of exo+ext-treated M2 microglia increased. The authors should quantify the IHC results to be able to make this statement (line 221).

6. Line 245: specify the fold change number if miRNA-148a-3p expression between Con and CoR to be more specific instead of saying it is "more obvious," which is a subjective statement.

Lines 231-233: in the figure legend, indicates "protein and gene expression" for immunofluorescence, western blotting, and qRT-PCR. Only qRT-PCR detects gene expression and does not detect protein, so this section should be edited appropriately.

7. Line 229: figure legend title is not accurate. Astrocytes were TBI-injured and then taken up by microglia. Consider re-writing to something like "TBI-injured astrocyte-derived exosomes promote the activation of microglia"

8. Line 250: where are the "necrotic and edematous brain tissues" from? Are these the brains from 7 days after TBI, or the extracted tissue from 1 day after TBI? Please clarify. The methods section only lists PCR use for microglia, not astrocytes. If astrocytes were examined this needs to be included in the methods.

9. The authors use several terms that are apparently interchangeable (eg. Con vs CoR; sham vs TBI; Con+Exo vs Ext+Exo; mimic vs agomer; inhibitor vs antagomir). This gets to be confusing for the reader. The authors should define these terms clearly in the methods and be consistent when possible. qRT-PCR (line 232) and RT-qPCR (line 250) are used interchangeably. Please stick with one throughout the manuscript
